# PPARG and the PTEN-PI3K/AKT Signaling Axis May Cofunction in Promoting Chemosensitivity in Hypopharyngeal Squamous Cell Carcinoma

**DOI:** 10.1155/2024/2271214

**Published:** 2024-03-11

**Authors:** Boxuan Han, Jiaming Chen, Shaoshi Chen, Xixi Shen, Lizhen Hou, Jugao Fang, Meng Lian

**Affiliations:** Department of Otorhinolaryngology Head and Neck Surgery, Beijing Tongren Hospital, Capital Medical University, Beijing 100730, China

## Abstract

It has been demonstrated that PPARG may interact with the PTEN-PI3K/AKT pathway, contributing to its involvement in the chemotherapy treatment of hypopharyngeal squamous cell carcinoma (HSCC). However, the underlying mechanism remains largely unknown. In this study, gene expression profiles of 17 HSCC patients, comprising 8 chemotherapy-sensitive patients (CSP) and 9 chemotherapy-nonsensitive patients (CNSP), were collected and analyzed to investigate expression patterns, correlations, influencing factors of the PPARG-PTEN-PI3K/AKT pathway, and its role in regulating chemosensitivity. The results revealed significantly increased expression (*p* < 0.04) of AKT1, AKT2, AKT3, PIK3CA, PPARG, and PTEN in the CSP group compared to the CNSP group. Specifically, AKT2 exhibited significant overexpression in tumor tissue (*p* = 0.01), while AKT2, AKT3, PPARG, and PTEN displayed significant increases in normal tissue (*p* ≤ 0.04). Positive correlations (*R* ∈ [0.43, 0.71], *p* < 0.014) were observed between PIK3CA, AKT1, AKT2, AKT3, and PTEN, with AKT2, AKT3, and PTEN also showing significant correlations with PPARG (*R* ∈ [0.35, 0.47], *p* < 0.04). Age, gender, and disease stage had no influence on PPARG, PIK3CA, and PTEN expression, but they may affect AKT expressions. Pathway analysis revealed that PPARG may interact with the PTEN-PI3K/AKT signaling pathway, playing a crucial role in regulating chemosensitivity in the normal tissue microenvironment. Our results suggest that AKT1 and PIK3CA may be associated with chemosensitivity in HSCC tumor cells, while PPARG and PTEN might exhibit a correlation with a specific segment of the PI3K/AKT pathway, potentially influencing chemosensitivity in the normal tissue microenvironment of HSCC patients.

## 1. Background

Hypopharyngeal squamous cell carcinoma (HSCC) is a type of cancer that originates in the squamous cells lining the hypopharynx, the lower part of the pharynx, or throat. It is a relatively rare but aggressive form of head and neck cancer [1]. Owing to its location and complex anatomy, HSCC often remains undetected until it reaches an advanced stage. Approximately 70-85% of HSCC patients receive their diagnosis at stage III or IV, making treatment more challenging [2].

Treatment options for HSCC typically depend on the stage and extent of the cancer but may include surgery, radiation therapy, chemotherapy, or a combination of these approaches [3]. However, the effectiveness of this approach in patients with HSCC is hindered by the potential development of chemotherapy resistance, which could significantly restrict clinical outcomes and impede improvements in survival rates [4].

Previous studies collectively suggest that the peroxisome proliferator-activated receptor gamma (PPARG) interacts with the PTEN-PI3K/AKT signaling pathway, playing a crucial role in regulating chemosensitivity in various cancers, including HSCC [5–8]. One study discovered that PPARG expression levels were slightly downregulated in chemotherapy-sensitive HSCC patients, indicating a potential association between PPARG and chemosensitivity [9]. Another study proposed that the upregulation of PPARG has been demonstrated to enhance the chemosensitivity of HSCC tumor cells by influencing both cell proliferation and cell motility pathways [5]. Polymorphisms in *cis*-regulatory elements of PTEN have been linked to heightened chemosensitivity in breast cancer [10]. Additionally, the PI3K/AKT pathway has been identified as a pivotal regulator of chemosensitivity. Genetic variations in Akt1, Akt2, and PIK3CA have been identified as being associated with chemosensitivity in cervical cancer patients [6]. Furthermore, andrographolide, a potential therapeutic agent in gastric cancer, deactivates the HIF-1 signaling pathway by inhibiting the upstream PI3K-AKT pathway. This suggests a correlation between chemosensitivity and PIK3CA [11]. Likewise, the lncRNA DUXAP8/miR-29a/PIK3CA network has been associated with chemotherapy resistance in B-cell acute lymphoblastic leukemia [12].

Therefore, it is logical to hypothesize that the PPARG and PI3K/AKT pathway, encompassing AKT1, AKT2, and PIK3CA, may potentially be associated with chemosensitivity in HSCC. Gaining insights into the expression patterns of these genes in both tumor and normal cells is essential for enhancing chemosensitivity while minimizing side effects on normal cells in HSCC treatment.

In this research, we analyzed the expression profiles of PPARG and key player genes within the PTEN-PI3K/AKT pathway to investigate their potential correlation with HSCC chemosensitivity. Furthermore, we established functional pathways to elucidate the potential mechanisms underlying this association. Our findings offer new insights into the impact of these genes in tumor cells and the normal tissue microenvironment associated with HSCC chemosensitivity, potentially contributing to the development of therapeutic strategies for treating HSCC.

## 2. Materials and Methods

### 2.1. Patient Recruit and Specimen Selection

A total of 17 patients diagnosed with HSCC between June 2012 and November 2017 at the Department of Head and Neck Surgery, Beijing Tongren Hospital, were included in this study. Among them, 8 patients were found to be sensitive to chemotherapy (CSP), while 9 patients were identified as nonsensitive to chemotherapy (CNSP). The treatment for all patients consisted of two cycles of chemotherapy using TPF (taxane/cisplatin/5-FU). The response to treatment was evaluated using RECIST 1.1 criteria. Surgical resection of the tumor was performed for each patient, and tissue specimens were collected from both the tumor and surrounding normal tissue. The surrounding normal tissue of the HSCC tumor was recognized through visual examination during the surgical resection. The criteria for choosing normal tissue involved ensuring the absence of macroscopic tumor infiltration, as assessed by the operating surgeon. The selection process aimed to reduce the risk of potential contamination from tumor cells. Information regarding gender, age, imaging examination results, blood routine tests, and biochemical tests was collected for all patients. Each tissue sample was rapidly frozen in liquid nitrogen and stored at -80°C. Ethical approval for the study was obtained from the Ethics Committee of Beijing Tongren Hospital (document number: TREC2023-KYS203), and written consent was obtained from all participants.

### 2.2. RNA Extraction, cDNA Synthesis, and In Vitro Transcription

TRIzol (Invitrogen) was employed to extract mRNA from tissue samples. The RNA quantity was determined through denaturing gel electrophoresis, revealing two distinct bands corresponding to 28S and 18S ribosomal RNA. This observation indicates the absence of DNA contamination or RNA degradation. To proceed, first-strand cDNA was synthesized using reverse transcription, followed by the conversion of single-stranded cDNA into double-stranded DNA using the PrimeScript™ Double Strand cDNA Synthesis Kit (TAKARA). Subsequently, the double-stranded DNA was purified to eliminate RNA, primers, and enzymes, serving as a template for in vitro transcription of biotinylated cRNA. Finally, the biotinylated cRNA was purified and prepared for hybridization with a microarray that had been prepared in advance.

### 2.3. Acquirement of mRNA Expression Profiles of HSCC

The mRNA expression profile of HSCC was analyzed using the Illumina Human HT-12 Bead Chip for hybridization with labeled cRNA. This microarray comprises 887 probes and six types of internal parameters to ensure sample quality control. The process involved incubating cRNA samples with the Illumina Human HT-12 Bead Chip at room temperature, followed by high-temperature and ethanol washes, and three additional room temperature washes. Subsequently, images were captured using the Illumina Bead Chip Reader software. To refine the raw data, the Illumina Genome Studio-Gene Expression software was utilized to filter background noise and handle missing values. Normalization was performed using the quantile method, and the gene expression profile was generated using the Illumina Custom software.

### 2.4. Analysis of Target Gene Expression

We employed one-way ANOVA to compare the gene expression profiles of PPARG and eight key genes in the PTEN-PI3K/AKT pathway in both normal and tumor tissues in response to chemotherapy. These genes encompassed AKT1, AKT2, AKT3, PIK3CA, PIK3CB, PIK3CD, PIK3CG, PPARG, and PTEN. Furthermore, we performed a coexpression analysis to explore the potential functional connections among these genes, employing Pearson's correlation coefficients (*R*) for evaluation. Both the *R*-value and associated *p* value were documented. In this context, we regard *p* values less than 0.05 and *R*-values greater than or equal to 0.6 as indicative of a strong correlation. Values within the range of 0.4 to 0.6 are considered to signify a moderate correlation, while values below 0.4 but greater than 0.3 suggest a weak correlation. Correlations below 0.3 are considered negligible. We utilized all available data, encompassing both tumor and normal tissue samples, to investigate the expression correlations among the nine genes under study. Additionally, a multiple linear regression (MLR) analysis was conducted to explore the potential impact of various factors, such as age, gender, and disease stage, on gene expression patterns. The analysis included reporting *p* values and *b*-values. In this context, *p* values below 0.05 were considered statistically significant. *p* values ranging from 0.05 to 0.1 were referred to as having “moderate statistical significance,” signifying a certain level of statistical importance. A *b*-value < 0 indicated a negative relationship, while a *b*-value > 0 suggested a positive relationship. The MLR analysis incorporated all available data, encompassing both tumor and normal tissue samples.

### 2.5. Functional Pathway Construction

The AI-based LDM was performed using the “AIC search” tool provided by AIC LLC (https://www.gousinfo.com/en/advancedsearch.html). This tool utilizes a vast dataset of 35 million citations and abstracts from the biomedical literature available on PubMed, along with protein-protein interaction data from STRING (https://string-db.org/).

Specifically, for genes that exhibited significant expression changes only in the normal tissue surrounding the HSCC tumor (including PPARG, AKT2, AKT3, and PTEN), the focus of the AI-based LDM was on understanding their impact on the tissue microenvironment associated with chemosensitivity. The goal was to uncover knowledge-database-supported relationships between these genes, various aspects related to tissue microenvironment regulation (such as apoptosis, cell cycle regulation, cell proliferation, cellular signaling, inflammatory response, drug metabolism, drug detoxification, and DNA repair), and their roles in promoting chemosensitivity.

Additionally, we conducted a subsequent manual review to ensure the quality and accuracy of the information retrieved through the AI-based LDM. These enhancements aim to provide a clearer understanding of our methodology for AI-based literature data mining.

## 3. Results

### 3.1. Clinical Data of the HSCC Patients

Table 1 provides a concise overview of key clinical data related to the HSCC patients enrolled in this study. Seventeen patients diagnosed with HSCC were analyzed. The majority of patients were male (88.24%), and their age was below 60 years (70.59%). Most cases were at stage IV (70.59%), with a smaller percentage at stage III (23.53%) and stage II (5.88%). Lymph node involvement (*N*) was observed in 52.94% of cases, while 29.41% had no lymph node involvement. All patients had no distant metastasis (M0). A response to induction chemotherapy was observed in 47.06% of cases, while 52.94% showed no response to treatment.

### 3.2. Gene Expression of PPARG and Key Players of PTEN-PI3K/AKT

**Figure 1:**
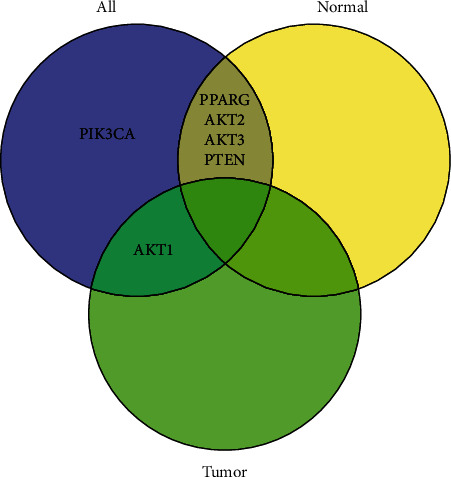
Significant genes in different comparisons. “Normal” represents the comparison using normal tissue surrounding the tumor. “Tumor” represents comparison using data from tumor tissue. “All” represents using both normal and tumor tissue.

In comparison of the CSP and CNSP groups, our analysis showed that six out of the nine genes demonstrated significantly increased expression using both normal and tissue data (*p* < 0.040), including AKT1, AKT2, AKT3, PIK3CA, PPARG, and PTEN (see Figure 1). However, only one gene, AKT2, presented a significant increase in tumor tissue (*p* = 0.01), and four genes showed a significant increase in normal tissue (AKT2, AKT3, PPARG, and PTEN; *p* ≤ 0.04). PIK3CA was the only gene that showed no significant change in normal or tumor tissue. We listed the details of the stats in Table 2, including the *p* value from one-way ANOVA analysis, the mean, and standard deviation (std) of effect size in terms of log fold change (LFC), which is a logarithmic transformation of the fold change, representing the ratio of gene expression levels between two conditions. A positive LFC indicates an increase in expression, while a negative LFC indicates a decrease.

### 3.3. Coexpression Analysis

Interestingly, the coexpression analysis revealed that PIK3CA exhibited a moderate to strong positive correlation (*R* ∈ [0.47, 0.66], *p* < 0.01) with AKT1, AKT2, AKT3, and PTEN, but displayed a relatively weak correlation with PPARG. PPARG demonstrated a weak to moderate correlation with AKT2, AKT3, and PTEN (*R* ∈ [0.35, 0.47], *p* < 0.04). It is not surprising that AKT1, AKT2, AKT3, and PTEN showed a moderate to strong mutually positive expression (*R* ∈ [0.42, 0.71], *p* < 0.01), as illustrated in Table 3. In this context, *R* refers to Pearson's correlation coefficients.

Our results indicate that the interaction between PPARG and PTEN-PI3K/AKT primarily involves the positive regulation of AKT2, AKT3, and PTEN in the normal tissue microenvironment, influencing the chemosensitivity of HSCC.

### 3.4. MLR Analysis Results

Table 3 presents the results of the MLR analysis, showing the *p* values for the variables age, stage, and gender for each gene (AKT1, AKT2, AKT3, PIK3CA, PPARG, and PTEN). The *p* values represent the statistical significance of the respective variables (age, stage, and gender) in relation to the expression of each gene. A smaller *p* value indicates a stronger statistical association between the variable and the gene's expression.

As shown in Table 4, the MLR analysis indicates that for some genes (e.g., AKT1, AKT2, and AKT3), age and gender may present statistically significant associations with gene expression (see the cells in italics) while for others (e.g., PIK3CA, PPARG, and PTEN), there are no statistically significant associations with the variables. Stage seems to have some moderate statistical significance in relation to AKT2, AKT3, and PTEN expression.

### 3.5. PPARG-Driven Functional Pathway Influencing Chemosensitivity

**Figure 2:**
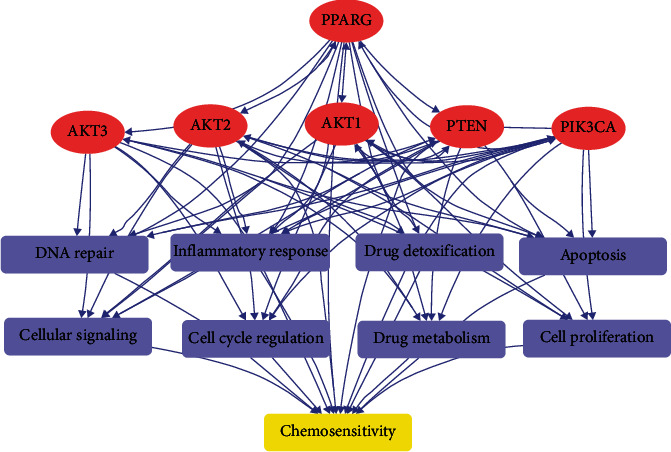
PPARG-driven functional pathway demonstrating its interaction with PTEN-PI3K/AKT signaling pathway regulating tissue microenvironment to influence chemosensitivity.

Literature-based pathway analysis reveals the crucial involvement of the PTEN-PI3K/AKT signaling pathway in regulating chemosensitivity, with PPARG playing a significant role in modulating the expression of AKT1, AKT2, AKT3, and PTEN. These findings present promising therapeutic opportunities for chemotherapy in HSCC, as supported by over 340 scientific publications (Figure 2).

The main finding of the pathway analysis highlights how gene expression in normal cells can impact chemosensitivity through various mechanisms, such as drug metabolism, detoxification, DNA repair, cell cycle regulation, apoptosis, inflammation response, and cellular signaling pathways. Multiple studies confirm that PPARG, a key regulator in several signaling pathways, exerts a notable influence on AKT1, AKT2, AKT3, and PTEN. Hence, PPARG and the PTEN-PI3K/AKT signaling pathway likely play crucial roles in regulating chemosensitivity by affecting drug metabolism, DNA repair, cell cycle regulation, and inflammatory responses. Consequently, targeting PPARG and PTEN-PI3K/AKT could hold great potential for enhancing chemotherapy efficacy across different cancer types, warranting further investigation in cancer therapy.

Despite not being explicitly centered on HSCC, our pathway analysis, in conjunction with the examination of HSCC expression data, revealed potential insights into how PPARG and PTEN-PI3K/AKT may regulate the normal tissue microenvironment, thus impacting chemosensitivity in HSCC patients.

## 4. Discussion

Previous studies have reported on the impact of PPARG on chemosensitivity in HSCC [5, 9]. However, the specific underlying mechanism remains largely unknown. Additionally, the expression of PPARG in tumor tissue has shown significant variability in patients' response to chemotherapy. In this study, we demonstrate that in the CSP group, PPARG consistently exhibits increased expression in the normal tissue surrounding the HSCC tumor (LFC = 2.00 ± 0.31), while its expression in the tumor tissue displays significant variation (LFC = 0.41 ± 0.80). This suggests that PPARG might influence the chemosensitivity in HSCC by regulating the microenvironment of the normal tissue.

Interestingly, in the comparison of CSP/CNSP groups, the genes AKT2, AKT3, and PTEN also exhibit a significant increase in expression in normal tissue but not in tumor tissue. Moreover, their expression pattern shows a strong positive correlation with that of PPARG (*R* ∈ [0.35, 0.47], *p* < 0.044). Two other key players in the PI3K/AKT pathway, AKT1 and PIK3CA, demonstrated significant overexpression in the tumor and overall, respectively, indicating their more direct role in regulating chemosensitivity in tumor cells rather than the normal tissue microenvironment. However, both AKT1 and PIK3CA displayed a statistically significant positive correlation with AKT2, AKT3, and PTEN, suggesting functional linkage in the regulation of HSCC chemosensitivity. Overall, our findings indicate that the PI3K/AKT pathway plays a crucial role in regulating HSCC chemosensitivity by influencing both tumor and normal cells to enhance chemosensitivity. On the other hand, PPARG and PTEN mainly interact with PI3K/AKT to play roles in the normal tissue microenvironment, influencing the chemosensitivity of HSCC.

It is important to note that our MLR analysis highlights that age, gender, and disease stage do not significantly affect the expression of PPARG, PTEN, and PIK3CA. This suggests that these genes consistently play a role in regulating chemosensitivity in HSCC. However, AKTs are partially susceptible to some of these factors (see Table 3).

The PI3K/Akt signaling pathway has been shown to play a crucial role in HSCC [13]. Clinical trials have investigated the efficacy of PI3K/Akt modulating agents in HSCC treatment. One such agent is buparlisib, an oral pan-PI3K inhibitor, which has shown promising results in phase II trials. Buparlisib targets the PI3K/Akt pathway and holds potential for therapeutic intervention in HSCC [13]. AKT2, AKT3, and PTEN could also exert influences on regulating multiple microenvironment-related factors that are associated with chemosensitivity, including drug metabolism, DNA repair, cell cycle regulation, inflammatory responses, and cellular signaling pathways, all contributing to chemosensitivity [14–18]. These findings suggest that targeting the PI3K/Akt pathway with modulating agents like buparlisib may be a promising strategy for HSCC treatment, potentially improving patient outcomes. Further research and clinical trials are warranted to fully evaluate the efficacy and safety of these agents in HSCC.

Our experimental data analysis results are supported by our pathway analysis, which reveals that PPARG interacts with part of the key players in the PTEN-PI3K/AKT pathway to regulate the normal tissue microenvironment, thus influencing chemosensitivity in cancers. In various in vitro and in vivo studies, there is consistent evidence supporting direct interactions between PPARG and key proteins in the PTEN-PI3K/AKT signaling pathway. Fructus Choerospondiatis (FC) components were found to regulate the PPAR signaling pathway, indicating a potential relationship between PPARG and AKT1 in the treatment of coronary heart disease [8]. Additionally, studies involving interventions such as RES, Zhenwu decoction, Chinese angelica (CHA), and Fructus aurantii (FRA) all highlight PPARG and AKT1 as common targets, emphasizing their potential therapeutic significance in various conditions including diabetic kidney disease and colorectal cancer [19–21].

Furthermore, investigations into specific compounds, such as kaempferol and quercetin, suggest their role in modulating PPARG expression and interacting with AKT1, pointing towards potential treatment avenues for endometrial cancer and nonsegmental vitiligo [22, 23]. The relevance of these interactions extends to conditions like uric acid nephropathy, pneumonia, and type 2 diabetes mellitus, as demonstrated by studies using Xiezhuo Huayu Yiqi Tongluo Formula (XHYTF), Fritillariae thunbergii bulbus, and Alpinia officinarum Hance [24–26].

The interplay between PPARG and AKT1 is also evident in the context of cancer, with studies identifying their association in colorectal cancer, acute myeloid leukemia, and breast cancer [27–29]. Additionally, the involvement of PPARG in cellular signaling pathways related to COVID-19 severity and hepatolenticular degeneration underlines its significance beyond traditional signaling networks [30, 31].

These studies collectively emphasize the direct interactions between PPARG and key proteins in the PTEN-PI3K/AKT signaling pathway across various physiological and pathological conditions, providing a foundation for further exploration of their therapeutic implications.

Pathway analysis also showed that PPARG significantly impacts chemosensitivity by regulating key cellular processes and signaling pathways involved in cancer cell response to chemotherapy. Its influence includes modulating apoptosis and cell survival, affecting autophagy [14] and chemoresistance. Propranolol, a nonselective betablocker, enhances chemosensitivity by promoting apoptosis in colorectal carcinoma cells [32]. A two-in-one nanoprodrug improves chemosensitivity in prostate cancer cells through increased apoptosis and metastasis inhibition [33]. PPARG also contributes to cell cycle dysregulation, related to chemosensitivity [34, 35], and influences drug detoxification processes, affecting chemotherapy efficacy [36, 37]. Moreover, PPARG's involvement in the inflammatory response is critical for determining chemosensitivity [38, 39].

We would like to highlight the following limitations. Firstly, our study primarily relies on gene expression analysis and bioinformatics approaches, lacking direct experimental validation of the proposed interactions. Experimental verification through cell culture models, animal studies, or functional assays is essential to establish causal relationships. Secondly, the study's retrospective nature and the relatively small sample size of 17 HSCC patients may limit the generalizability of our findings. Future studies with larger cohorts are warranted to validate and extend our observations. Additionally, the lack of functional assays and mechanistic experiments constrains the depth of our insights into the precise molecular mechanisms. Despite these limitations, our study lays the groundwork for further investigations into the intricate molecular pathways influencing chemosensitivity in HSCC.

Acknowledging the limitations of our study, future research endeavors should prioritize several key aspects. (1) Experimental validation is crucial to confirm the interactions and regulatory roles proposed in our study, necessitating further experiments in cell culture models, animal studies, or functional assays. (2) Extending our findings to larger clinical cohorts for validation and correlating gene expression patterns with treatment outcomes will enhance clinical relevance. (3) Identifying therapeutic targets within the PPARG-PTEN-PI3K/AKT pathway is vital, urging the exploration of targeted therapies to modulate these pathways and improve chemosensitivity. (4) Integrating multiomics data, encompassing genomics, proteomics, and epigenomics, can provide a comprehensive understanding of the molecular landscape underlying chemosensitivity in HSCC, potentially revealing additional regulators and pathways. (5) Additionally, in-depth mechanistic studies delving into the specific molecular mechanisms by which PPARG and other key genes influence the tumor microenvironment and chemosensitivity should be pursued. These avenues of investigation aim to address the complexities of HSCC chemosensitivity comprehensively.

## 5. Conclusion

According to our findings, there is a correlation suggesting that AKT1 and PIK3CA may be associated with chemosensitivity in HSCC tumor cells, while PPARG and PTEN might exhibit a correlation with a specific segment of the PI3K/AKT pathway, potentially influencing chemosensitivity in the normal tissue microenvironment of HSCC patients.

## Figures and Tables

**Table 1:** Characteristics of 17 diagnosed hypopharyngeal squamous cell carcinoma patients.

Characteristics	*N* (%)
Sex	
Male	15 (88.24%)
Female	2 (11.76%)
Age (year)	
≤60	12 (70.59%)
>60	5 (29.41%)
Stage	
I	0 (0%)
II	1 (5.88%)
III	4 (23.53%)
IV	12 (70.59)
T	
1	0 (0%)
2	3 (17.65%)
3	4 (23.53%)
4	10 (58.82%)
N	
0	5 (29.41%)
1	2 (11.76%)
2	9 (52.94%)
3	1 (5.88%)
M	
0	17 (100%)
Response to induction chemotherapy	
Response	8 (47.06%)
Nonresponse	9 (52.94%)

**Table 2:** Expression of nine genes compared between the CSP and CNSP groups.

Gene name	All			Normal			Tumor		
	*p* value	Mean LFC	Std LFC	*p* value	Mean LFC	Std LFC	*p* value	Mean LFC	Std LFC
AKT1	0.02	0.57	0.67	0.50	0.17	0.45	0.26	0.90	1.92
AKT2	0.01	0.66	0.34	0.02	0.67	0.21	0.01	0.97	0.39
AKT3	0.01	2.45	0.97	0.01	3.35	0.97	0.12	0.65	0.45
PIK3CA	0.04	0.76	0.76	0.09	0.80	0.56	0.23	1.56	0.96
PIK3CB	0.16	0.50	0.49	0.16	0.52	0.50	0.17	7.60	0.24
PIK3CD	0.34	0.46	1.41	0.53	0.51	1.90	0.16	0.73	0.71
PIK3CG	0.37	0.57	1.85	0.14	1.41	2.09	0.44	0.47	0.40
PPARG	0.01	1.45	1.66	0.010	2.00	0.31	0.49	0.41	0.80
PTEN	0.01	9.19	0.30	0.040	10.80	0.32	0.76	-0.30	1.59

Note. “Normal” represents the comparison using normal tissue surrounding the tumor. “Tumor” represents comparison using data from tumor tissue. “All” represents using both normal and tumor tissue.

**Table 3:** Coexpression analysis for the six genes shows significance in CSP vs. CNSP comparison.

	PPARG	AKT1	AKT2	AKT3	PTEN	PIK3CA
PPARG	/	-0.03|0.88	*0.41|0.02*	*0.35|0.04*	*0.47|0.00*	0.21|0.23
AKT1	-0.03|0.88	/	*0.59|0.00*	*0.46|0.01*	*0.42|0.01*	*0.66|0.00*
AKT2	*0.41|0.02*	*0.59|0.00*	*/*	*0.64|0.00*	*0.43|0.01*	*0.47|0.01*
AKT3	*0.35|0.04*	*0.46|0.01*	*0.64|0.00*	*/*	*0.71|0.00*	*0.63|0.00*
PTEN	*0.47|0.00*	*0.42|0.01*	*0.43|0.01*	*0.71|0.00*	*/*	*0.60|0.00*
PIK3CA	0.21|0.23	*0.66|0.00*	*0.47|0.01*	*0.63|0.00*	*0.60|0.00*	*/*

Note. The data is presented in the format “*R*-value|*p* value” for each cell in the table. Cells in italics represent statistically significant correlations.

**Table 4:** MLR analysis results.

	*p* value for age	*p* value for stage	*p* value for gender
AKT1	*0.03|0.04*	0.08|0.28	*0.65|0.04*
AKT2	*0.03|0.04*	0.20|0.07	0.39|0.14
AKT3	0.04|0.25	0.72|0.07	*2.28|0.05*
PIK3CA	-0.010.64	0.25|0.16	-0.15|0.61
PPARG	-0.05|0.90	0.20|0.30	0.67|0.24
PTEN	-0.08|0.63	2.95|0.08	3.83|0.26

Note. The data is presented in the format “*b*-value|*p* value” for each cell in the table. Cells in italics represent statistically significant influence.

## Data Availability

The data of this study are available from the corresponding authors upon reasonable request.
